# A Systematic Analysis on mRNA and MicroRNA Expression in Runting and Stunting Chickens

**DOI:** 10.1371/journal.pone.0127342

**Published:** 2015-05-26

**Authors:** Li Zhang, Ying Li, Xiujuan Xie, Haiping Xu, Zhenqiang Xu, Jinge Ma, Bixiao Li, Shudai Lin, Qinghua Nie, Qingbin Luo, Xiquan Zhang

**Affiliations:** 1 Guangdong Provincial Key Lab of Agro-animal Genomics and Molecular Breeding and Key Lab of Chicken Genetics, Breeding and Reproduction, Ministry of Agriculture, South China Agricultural University, Guangzhou, 510642, P.R. China; 2 Agricultural College, Guangdong Ocean University, Zhanjiang, 524088, P.R. China; 3 Institute of Animal Science, Guangdong Academy of Agricultural Sciences, Guangzhou, 510640, P.R. China; University of Louisville, UNITED STATES

## Abstract

Runting and stunting syndrome (RSS), which is characterized by lower body weight, widely occurs in broilers. Some RSS chickens simply exhibit slow growth without pathological changes. An increasing number of studies indicate that broiler strains differ in susceptibility to infectious diseases, most likely due to their genetic differences. The objective of this study was to detect the differentially expressed miRNAs and mRNAs in RSS and normal chickens. By integrating miRNA with mRNA expression profiling, potential molecular mechanisms involved in RSS could be further explored. Twenty-two known miRNAs and 1,159 genes were differentially expressed in RSS chickens compared with normal chickens (*P* < 0.05). qPCR validation results displayed similar patterns. The differentially expressed genes were primarily involved in energy metabolism pathways. The antisense transcripts were extensively expressed in chicken liver albeit with reduced abundance. Dual-luciferase reporter assay indicated that gga-miR-30b/c directly target *CARS* through binding to its 3′UTR. The miR-30b/c: *CARS* regulation mainly occurred in liver. In thigh muscle and the hypothalamus, miR-30b/c are expressed at higher levels in RSS chickens compared with normal chickens from 2 to 6 w of age, and notably significant differences are observed at 4 w of age.

## Introduction

Runting and stunting syndrome (RSS), which is characterized by low body weight, mainly occurs in broilers, and the condition is incredibly complicated [1–3]. Previous reports indicate that both genetic and environmental factors, such as diseases and feeding, are implicated in arrested development [4, 5]. The body weights of RSS chickens are significantly reduced compared with normal chickens. RSS generally occurs early in life and always results in considerable economic losses, particularly in the commercial broiler industry. Previous reports indicated that RSS chickens are frequently associated with viral infections, such as avian leukosis virus [6], reticulo endotheliosis virus [7], digestive tract disease [8], and astroviruses [3]. Currently, no effective commercial vaccine available for the control of this disease exists, primarily owing to the absence of known etiologic agents. In addition, some RSS chickens grow slowly without pathological changes. These chickens typically survive until sold. An increasing number of studies indicate that broiler strains differ in susceptibility to infectious diseases, most likely due to their genetic differences [9–11].

RNA sequencing (RNA-seq) technology holds great potential for disease diagnosis and genetic differences analyses [12]. So far, large-scale mRNA expression analysis in RSS chickens is lacking. Additionally, an increasing number of reports indicate that microRNAs (miRNAs), such as let-7b, miR-1, miR-133 and miR-206 [13, 14], function in animal growth by regulating their target genes. Therefore, an integrative analysis of miRNA and mRNA interaction in RSS chickens was conducted to investigate the genetic differences between RSS and normal chickens and identify potential markers for growth.

In the present study, we identified differentially expressed miRNAs and mRNAs. These genes were mainly enriched in energy metabolism pathways, and these genes could serve as candidate genes for RSS chickens. The data from this study provide a combined analysis of miRNAs and mRNAs and shed new light on the fine-tuning process of growth.

## Results

### 2.1 RSS chicken classification

**Fig 1:**
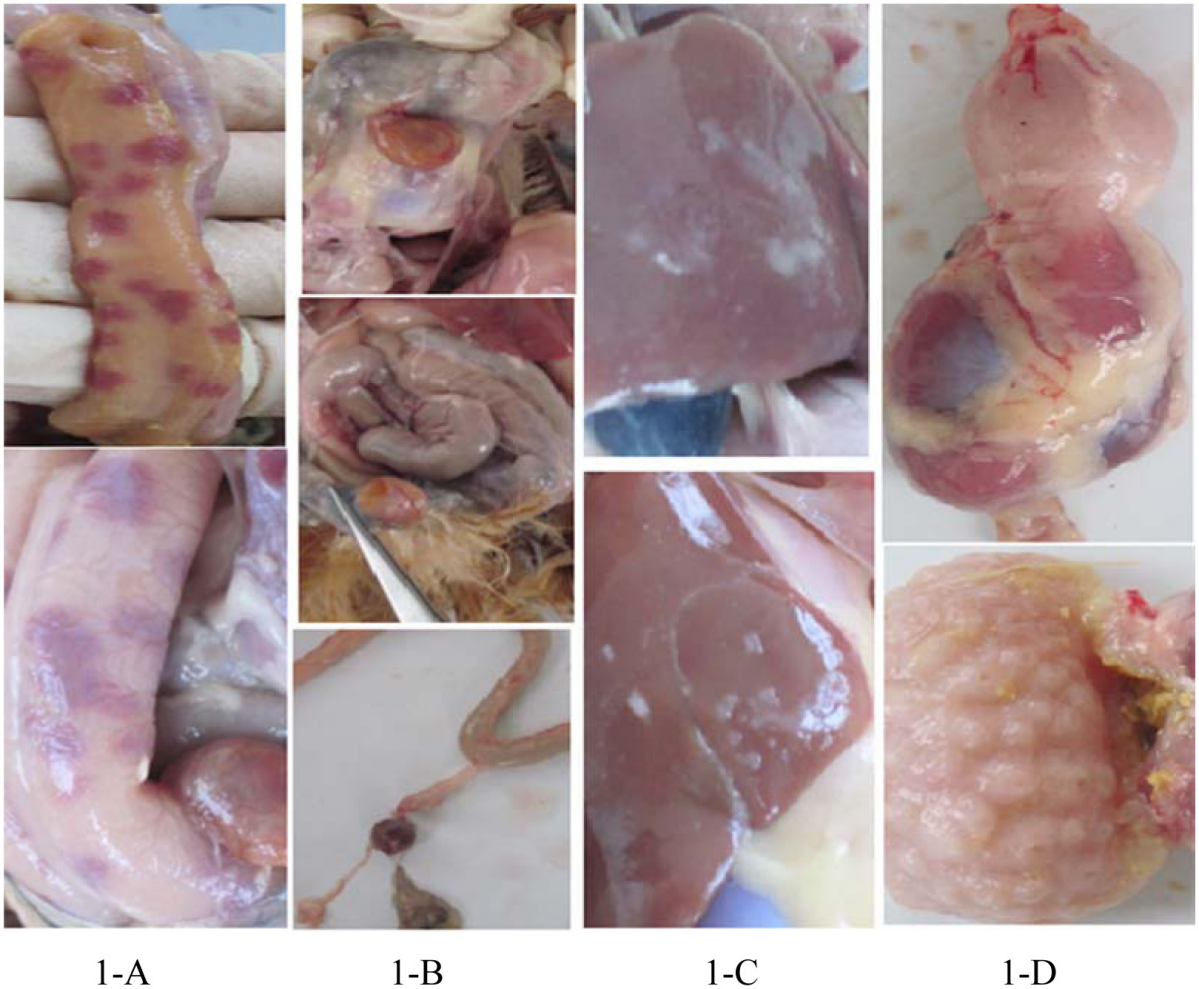
Pathologic changes of RSS chicken. 1-A, intestinal bleeding; 1-B, yolk malabsorption; 1-C, perihepatitis; 1-D, proventriculitis.

**Fig 2:**
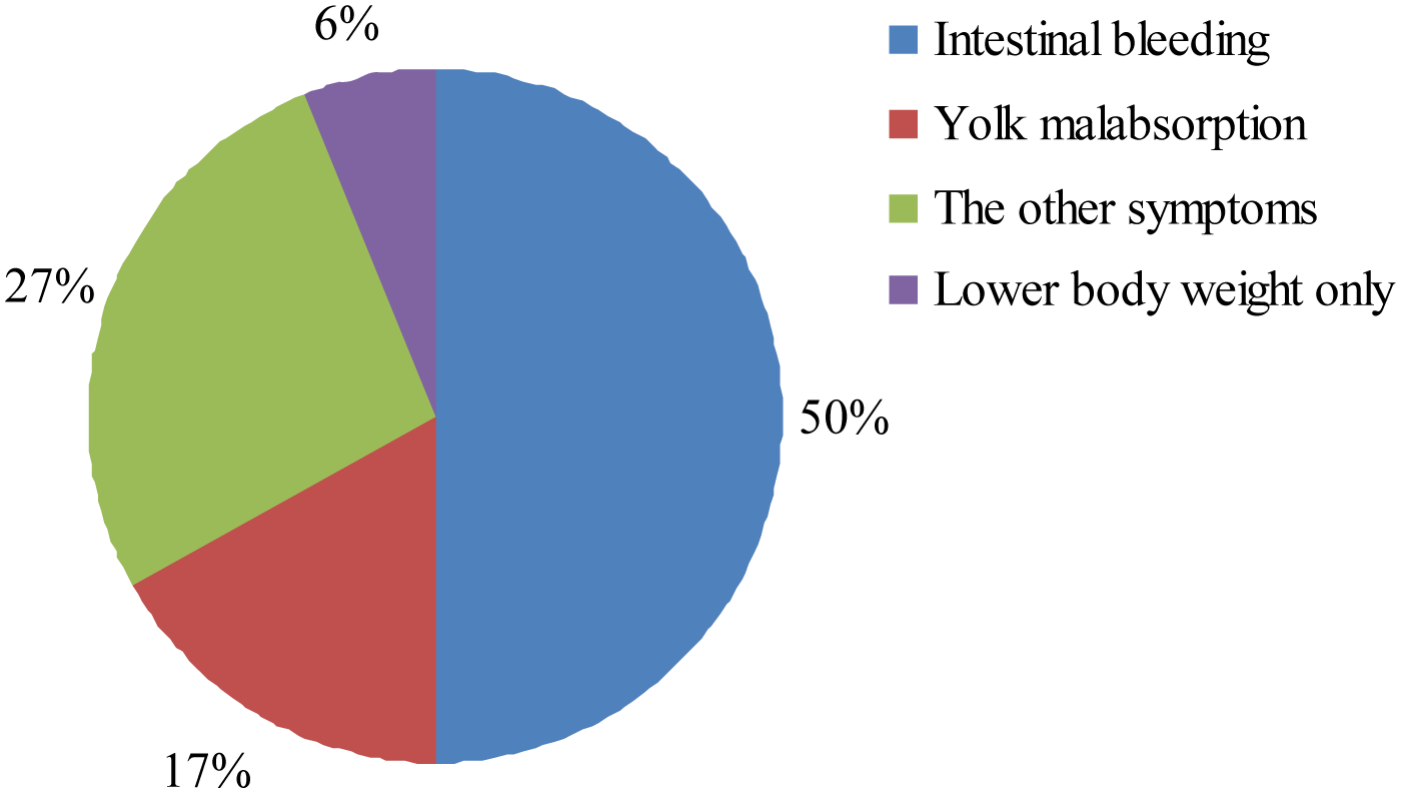
The types and incidences of RSS chicken.

In the experiment, we dissected a total of 400 RSS chickens and 24 (6%) of them exhibited no pathological changes except for lower body weight. The remaining chickens showed various symptoms, such as intestinal bleeding, yolk malabsorption, perihepatitis and proventriculitis (Figs 1 and 2). All the 24 RSS individuals were ALV and REV virus negative. Five negative RSS chicken and 5 normal healthy individuals were used for Solexa and Digital Gene Expression (DGE) sequencing.

### 2.2. Differentially expressed miRNAs between the livers of RSS and normal chickens

**Fig 3:**
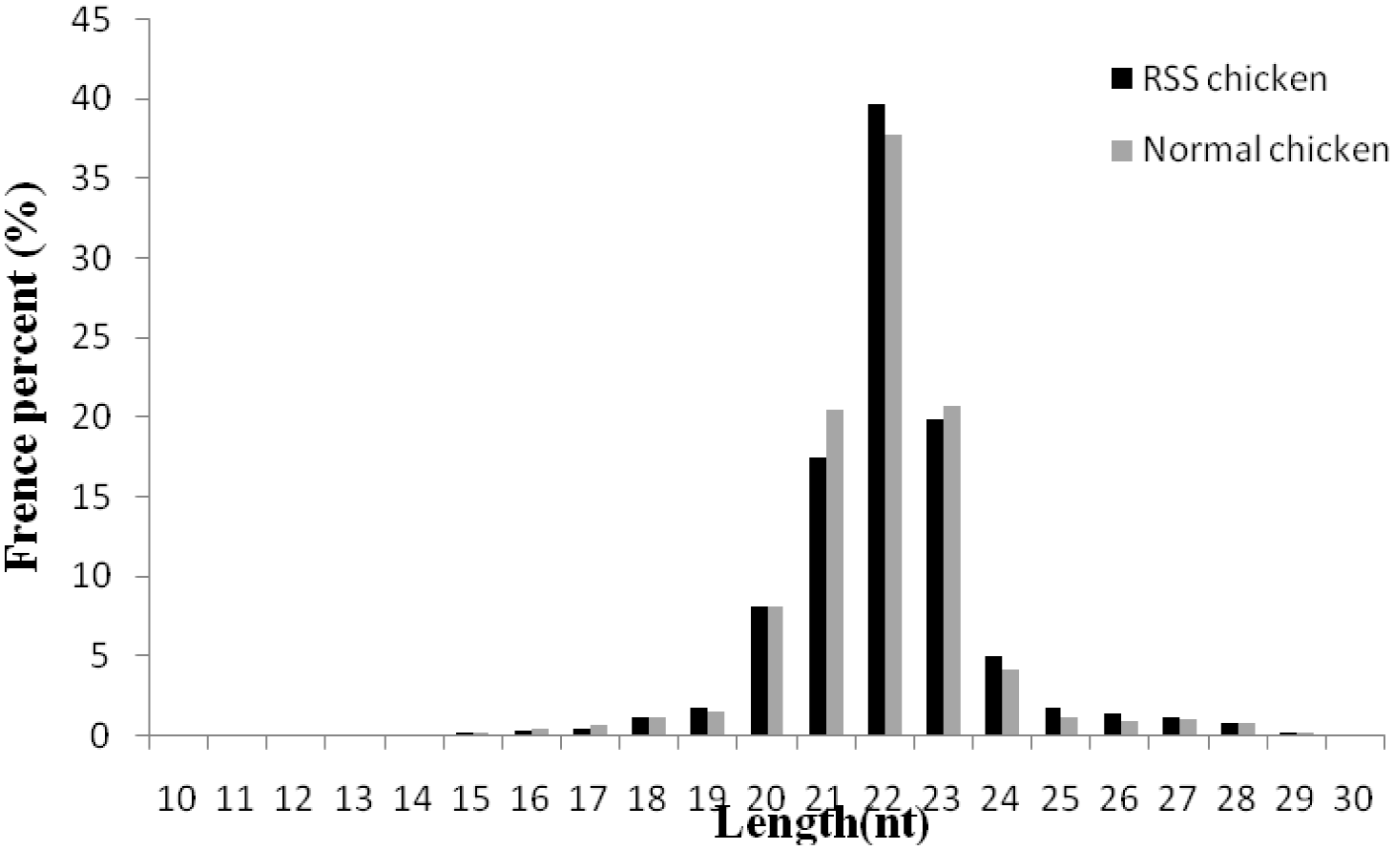
Length distribution of small RNA after quality trimming and adaptor removal.

From Solexa miRNA sequencing, we obtained a total of 9,246,256 and 8,714,768 small RNA high quality reads in RSS and normal chicken livers, respectively. Length distribution analysis showed that most reads ranged from 21–23 nt with the percentage of the three types of reads being 77.18% and 79.10% for the two libraries, respectively (Fig 3). The high quality reads were subsequently annotated to different classes of RNA categories using different databases such as miRBase (V19.0), from Rfam (10.1) and Genbank to identify miRNAs, repeats-associated RNA, rRNA, tRNA, snRNA, snoRNA, etc. In total, 6,246,916 and 6,002,911 reads were annotated and the most abundant RNA species in the two libraries was classified as miRNAs (Table 1).

**Table 1:** Distribution of the genome-mapped sequence reads in small RNA libraries.

	Locus class		Normal chickens	
RSS chickens	Unique sRNA	Total reads	Unique sRNA	Total reads
exonantisense	527	630	529	622
exonsense	48,526	72,380	54,961	76,859
intronantisense	3,915	8,587	3,743	7,457
intronsense	13,875	94,228	11,140	79,634
microRNA	2,246	5,125,708	2,098	5,228,294
rRNA	53,750	785,596	39,632	481,979
repeat	7,832	11,684	6,533	9,150
scRNA	1,145	9,373	934	8,484
snRNA	2,133	9,990	1,551	5,877
snoRNA	3,553	28,384	2,413	23,391
tRNA	9,281	100,356	7,635	81,164
unannotated reads	328,735	2,999,340	32,6472	2,711,857

In this study, 202 and 194 known miRNAs were detected in RSS and normal chickens, respectively. Among these known miRNAs, 22 were differentially expressed (*P* < 0.05 and Fold-change ≥1) (Table 2). Four up-regulated miRNAs (gga-miR-221, gga-miR-30b, gga-miR-30c and gga-miR-215) and 1 down-regulated miRNA (gga-miR-375) in RSS chicken were chosen randomly to validate the Solexa sequencing results using RT-qPCR. Our results indicated that the miRNA expressions displayed patterns similar to those observed using Solexa analysis (Table 3).

**Table 2:** miRNAs differentially expressed in the livers of RSS and normal chickens.

					Fold-change(|log2 TPM Normal/TPM RSS|)		
miRNA	Cou-RSS	Cou-Normal	TPM-RSS	TPM-Normal	Up-regulated in RSS chickens	Up-regulated in normal chickens	*P*
miR-196	4	24	0.43	2.75		2.67	<0.0001
miR-499	4	21	0.43	2.41		2.48	0.0003
miR-216b	5	24	0.54	2.75		2.35	0.0002
miR-1551	10	34	1.08	3.9		1.85	0.0001
miR-217	12	34	1.3	3.9		1.59	0.0005
miR-375	556	1249	60.13	143.32		1.25	<0.0001
miR-1682	21	40	2.27	4.59		1.02	0.0078
miR-30c	931	414	100.69	47.51	1.08		<0.0001
miR-221	1023	452	110.64	51.87	1.09		<0.0001
miR-2188	201	88	21.74	10.1	1.11		<0.0001
miR-30b	217	91	23.47	10.44	1.17		<0.0001
miR-215	1305	547	141.14	62.77	1.17		<0.0001
miR-1662	1534	636	165.91	72.98	1.18		<0.0001
miR-18b	25	10	2.7	1.15	1.24		0.01846
miR-3536	383	127	41.42	14.57	1.51		<0.0001
miR-23b	49	16	5.3	1.84	1.53		<0.0001
miR-3535	24	6	2.6	0.69	1.91		0.0015
miR-1744	42	9	4.54	1.03	2.14		<0.0001
miR-3524	11	2	1.19	0.23	2.37		0.0175
miR-367	12	0	1.3	0.01	7.02		0.0004
miR-302b	339	1	36.66	0.11	8.32		<0.0001
miR-302c	47	0	5.08	0.01	8.99		<0.0001

**Table 3:** RT-qPCR validation of miRNA expression (RSS *Vs*. Control).

	Fold-change (|log2 TPM RSS/TPM Normal|) (Solexa sequencing)		Fold-change (|log2 relative expression in RSS/in Normal|) (RT-qPCR)		
miRNA	Up-regulated in RSS chicken	Down-regulated in RSS chicken	Up-regulated in RSS chicken	Down-regulated in RSS chicken	Trends
gga-miR-221	2.13		3.21		identical
gga-miR-30b	2.25		2.34		identical
gga-miR-30c	2.12		2.31		identical
gga-miR-215	2.25		1.97		identical
gga-miR-375		2.38		2.46	identical

### 2.3. Differentially expressed genes between RSS and normal chicken livers

DGE-sequence obtained 3,768,716 and 3,632,492 high quality clean tags in RSS and normal chicken livers, respectively. In the obtained clean tags, 2,134,676 (56.64%) and 1,900,218 (52.31%) tags were mapped to 11,627 and 11,381chicken genes. Compared with normal chickens, a total of 1,159 genes (605 up-regulated and 554 down-regulated) were differentially expressed (*P* < 0.05 and Fold-change ≥ 1) in RSS chickens.

To verify the DGE sequencing results, two up-regulated genes (*CKAP2L* and *TBPL1)* and six down-regulated genes (*MAP2K1*, *KDR*, *CARS*, *MAP3K7IP2*, *MAP3K5* and *LARP7*) were selected for qPCR analysis. The qPCR results displayed patterns similar to DGE sequencing (Table 4). Of the differentially expressed sequences, six genes (*LOC415662*, *C1orf78*, *ZZEF1*, *CEBPA*, *LOC418836* and *FOXP2*) were unique in RSS chickens, whereas 5 genes (*CINP*, *MYH3*, *EEF1A2*, *MAP3K13* and *IMMP1L*) were unique in normal chickens. Several immune related genes, *MAP3K7IP2*, T-cell immune regulator 1 (*TCIRG1*), *MAP2K1*, *STAT5B*, *SOCS2*, *CSF1R*, *EXTL3* and *GAMT* were also differentially expressed in RSS versus normal chickens. Notably, *MAP3K7IP2* and *TCIRG1* are related to the T cell receptor (TCR) signaling pathway, which has important functions in animal immunity [15]. Both genes exhibited significantly different expression in RSS versus normal chickens (*P* < 0.001).

**Table 4:** RT-qPCR validation of DGE results (RSS *Vs*. Control).

	Fold-change (DGE) (|log2 TPM RSS/TPM Normal |)		Fold-change(RT-qPCR) (|log2 relative expression in RSS/in Normal|)		
Gene	Up-regulated in RSS chicken	Down-regulated in RSS chicken	Up-regulated in RSS chicken	Down-regulated in RSS chicken	Trends
*CKAP2L*	14.16		2.18		identical
*TBPL1*	7.67		1.7		identical
*MAP2K1*		4.6		1.03	identical
*KDR*		9.55		1.1	identical
*CARS*		2.11		1.48	identical
*MAP3K7IP2*		2.36		1.91	identical
*MAP3K5*		6.21		1.80	identical
*LARP7*		1.25		1.44	identical

**Fig 4:**
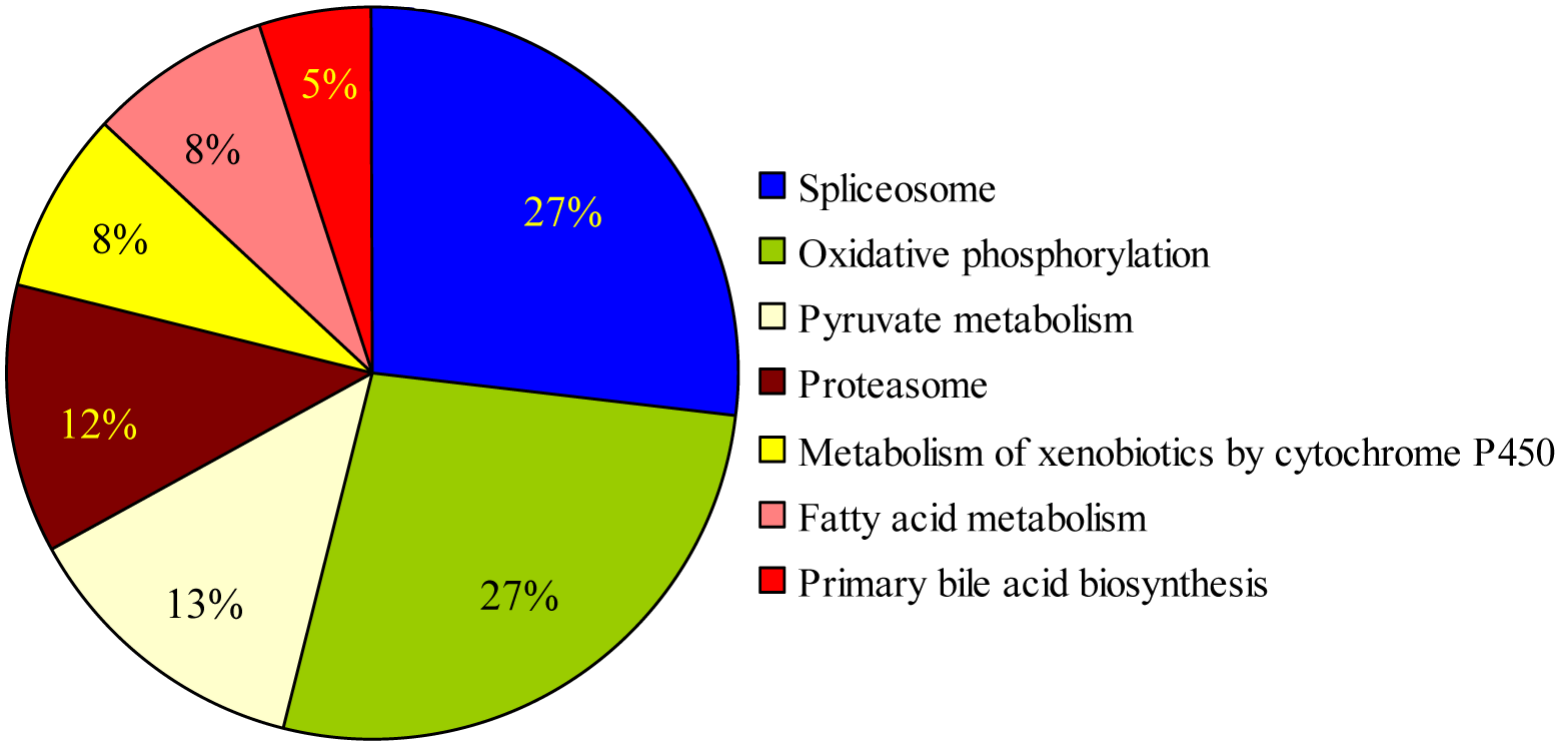
The distribution of matches to gene ontology categories. In total, 1159 differentially expressed genes, identified using the DAVID v6.7, MAS3.0 and BGI-Cloud annotation tools, were enriched in seven pathways.

To explore the biological meaning of the differentially expressed genes, DAVID v6.7 (http://david.abcc.ncifcrf.gov/), MAS3.0 (http://bioinfo.capitalbio.com/mas3/) and BGI-Cloud (http://bgiamericas.com/data-analysis/bioinformatics-software/) functional annotation tools were employed. Ninety-two known genes were successfully annotated with confident matches, and these genes were involved in 7 pathways, including fatty acid metabolism, oxidative phosphorylation, etc. (Fig 4).

### 2.4. Antisense transcription

In the present study, 7,237 genes in RSS and 7,363 genes in normal chickens were found to possess antisense transcripts, thereby indicating that the antisense transcripts were extensively expressed in chicken liver. The majority of the antisense tags were expressed in lower abundance than their corresponding sense tags. KEGG analysis of the genes with antisense tags implied that these genes were notably enriched in 15 pathways in RSS chickens and 17 pathways in normal chickens (*P* < 0.05, Table 5). The amino acyl-tRNA biosynthesis pathway was particularly enriched in RSS chickens, whereas DNA replication, RNA degradation and ubiquitin-mediated proteolysis pathways were enriched in normal chickens (Table 5).

**Table 5:** The involvement of antisense genes in pathways predicted by KEGG enrichment analysis.

		Unigenes	
No.	Pathway	RSS chickens	Normal chickens
1	Amino sugar and nucleotide sugar metabolism	20	21
2	Aminoacyl-tRNA biosynthesis	21	0
3	Butanoate metabolism	15	15
4	DNA replication	0	15
5	Fatty acid metabolism	17	19
6	Glycolysis / Gluconeogenesis	26	28
7	Glyoxylate and dicarboxylate metabolism	9	10
8	Limonene and pinene degradation	8	8
9	Lysosome	47	43
10	Porphyrin and chlorophyll metabolism	14	13
11	PPAR signaling pathway	31	31
12	Propanoate metabolism	18	18
13	Pyruvate metabolism	21	21
14	RNA degradation	0	24
15	Spliceosome	49	51
16	Starch and sucrose metabolism	14	14
17	Ubiquitin mediated proteolysis	0	51
18	Valine, leucine and isoleucine degradation	22	22

### 2.5. Integrative analysis of miRNA and mRNA expression data

The target genes of 22 differentially expressed miRNAs were predicted using Targetscan (http://www.targetscan.org/), Microcosm (http://www.ebi.ac.uk/enright-srv/microcosm/htdocs/targets/v5/info.html), miRDB (http://mirdb.org/miRDB/) and RNAhybrid (http://bibiserv.techfak.uni-bielefeld.de/rnahybrid/). The overlapping genes obtained from the four programs were considered candidate target genes. Consequently, 149 coordinated genes, which displayed differentially expressed mRNAs and were also potential targets of miRNAs, were identified (Table 6). Several genes, such as cysteinyl-tRNA synthetase (*CARS*), were related to development. CARS has various biological functions, such as *ATP* binding and tRNA binding. The human *CARS* gene is located on chromosome band 11p15.5, and chromosomal rearrangements in the region are associated with Silver-Russell syndromes [16] and Beck with-Wiedemann syndromes [17].

**Table 6:** The integrative differentially expressed miRNAs and potential target genes.

miRNA	Number	Potential target genes
gga-mir-18b	5	*CDC2L6*, *GYG1*, *MBNL1*, *MPP4*, *SAV1*
gga-mir-196	1	*GLO1*
gga-mir-215	3	*CTCF*, *CTNNBIP1*, *HPCAL1*
gga-mir-217	5	*FXR1*, *IARS*, *TMCO1*, *UMPS*
gga-mir-221	6	*EIF3J*, *GNPTAB*, *MAP3K7IP2*, *MIA3*, *MYLIP*, *PCMTD1*
gga-mir-23b	20	*ADNP*, *ALDH5A1*, *C4ORF29*, *CFL2*, *CTCF*, *DCUN1D5*, *DHX15*, *DIABLO*, *EIF4E3*, *ELF2*, *FADS1*, *FOXP2*, *HNRNPU*, *MAP3K8*, *NARG1*, *RBM25*, *SS18L2*, *TMPO*, *WTAP*, *ZEB1*
gga-mir-302b	6	*BAMBI*, *CDC2L6*, *CIRBP*, *KLHL2*, *TXNDC11*, *UBE2W*
gga-mir-302c	5	*BAMBI*, *CDC2L6*, *CIRBP*, *GPI*, *UBE2W*
gga-mir-30b	27	*ADRBK2*, *ALDH5A1*, *AMOTL2*, *ATP2B1*, *BECN1*, *CARS*, *CCDC6*, *CFL2*, *CHKA*, *CPSF33L L*, *DNAJC13*, *ERRFI1*, *ESCO1*, *GATM*, *HERC2*, *HIVEP1*, *ITPK1*, *JMJD1A*, *MMD*, *NT5C1B*, *PPID*, *RAPGEF4*, *RASA1*, *REV3L*, *SMARCA5*, *STK39*, *TXNDC5*
gga-mir-30c	31	*ADRBK2*, *AMOTL2*, *ATP2B1*, *BECN1*, *C6ORF115*, *CARS*, *CCDC6*, *CDCA7L*, *CFL2*, *CHKA*, *CPSF3L*, *DNAJC13*, *ERRFI1*, *ESCO1*, *GATM*, *HERC2*, *HIVEP1*, *ITPK1*, *JMJD1A*, *LYG2*, *MMD*, *NT5C1B*, *PPID*, *RAPGEF4*, *RASA1*, *RCJMB04_1P22*, *REV3L*, *SMARCA5*, *STK39*, *TXNDC5*
gga-mir-367	26	*C6orf62*, *CDCA7L*, *CHKA*, *CRYL1*, *DKK3*, *FAM135A*, *FXR1*, *GNS*, *HAND2*, *HERPUD2*, *HIVEP1*, *IQGAP2*, *MAN1B1*, *MIA3*, *MMD*, *MYLIP*, *PCMTD1*, *PLG*, *RAB14*, *RAD21*, *REV3L*, *RGS3*, *SETD5*, *STK39*, *UBE2W*, *UGP2*, *ZFYVE21*
gga-mir-375	3	*GPT2*, *LDHB*, *RNF126*
gga-mir-499	6	*DYNLT1*, *EML4*, *ERCC4*, *MAN1B1*, *PTBP2*, *RCJMB04_2h11*

### 2.6. gga-miR-30b/c directly target *CARS* through binding to its 3′UTR

**Fig 5:**
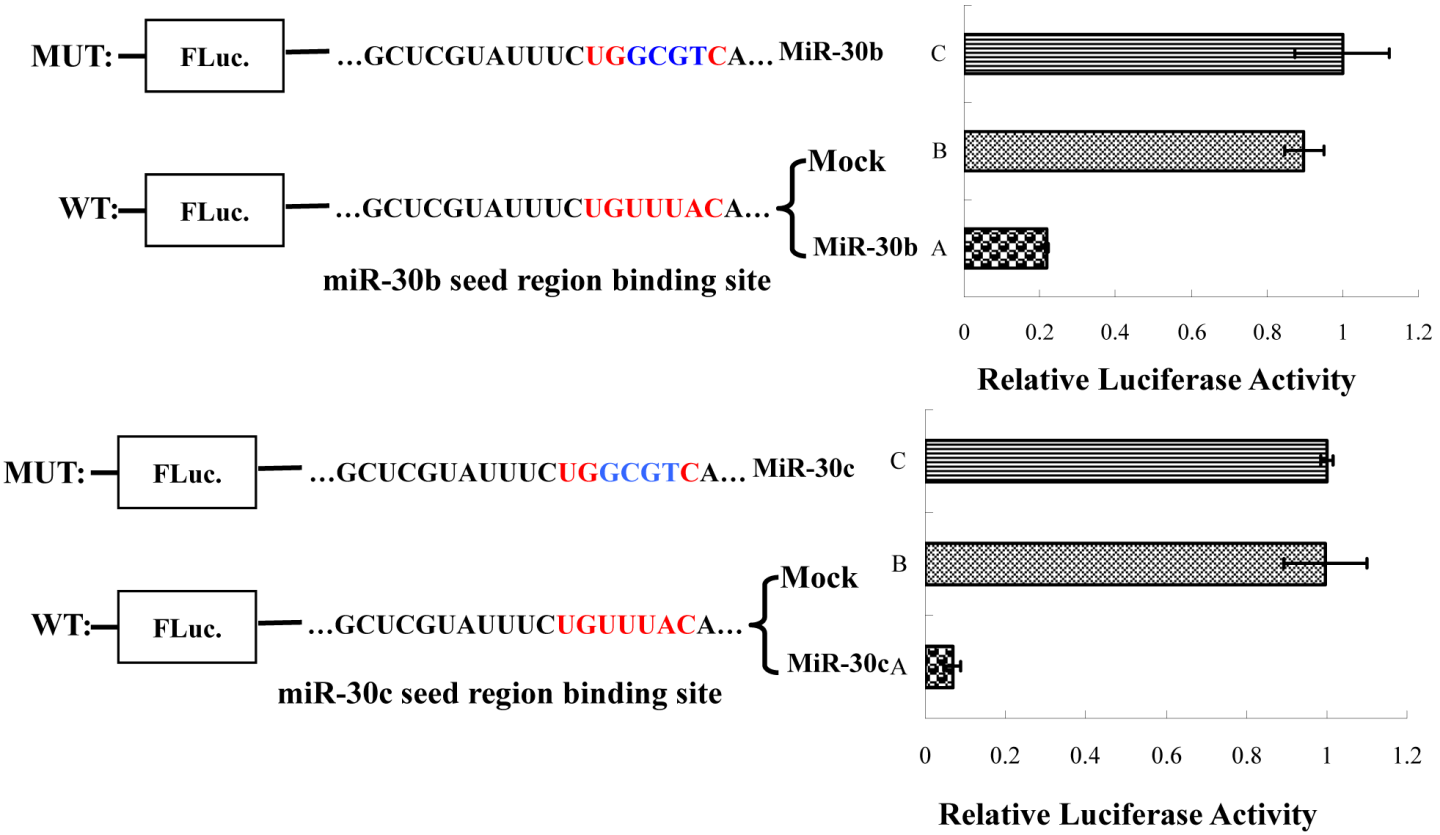
Dual-luciferase reporter assay for the 3′ UTR of *CARS* as the target site of miR-30b/c in vitro. pCDNA3.1-miR-30b/c plasmid were cotransfected with the pmirGLO-*CARS* 3′ UTR, pmirGLO- *CARS* 3′UTR mutation, or the control plasmid into DF-1 cells. The relative luciferase activities were measured.

Previous studies reported that gga-miR-30b/c were involved with growth and development [18–22]. The inhibitory relationship between gga-miR-30b/c and their predicted target gene *CARS* was verified using luciferase reporter assays. The wild-type and mutated target sequences of *CARS* 3′UTR were introduced into pmirGLO vector (Promega Biotech Co., Ltd., China), We co-transfected 200 ng pmirGLO-CARS or pmirGLO-CARS mutant, with 600 ng pCDNA3.1-miR-30b/c plasmid, into DF-1 cells. Thirty-six hours later, the cells were lysed, and the luciferase activity was measured using the Dual Luciferase Assay according to the manufacturer’s protocol (Promega Biotech Co., Ltd., China). Compared with the cells that were transfected with pmirGLO-CARS mutant, the luciferase activity of *CARS* 3′UTR wild-type was significantly decreased. Also, the luciferase activity of the mock transfection was higher than that of the wild-type group (Fig 5). These results indicated that *CARS* 3′UTRwas directly targeted by miR-30b/c.

### 2.7. Temporal and spatial expression of miR-30b/c and *CARS* gene in RSS chickens

**Fig 6:**
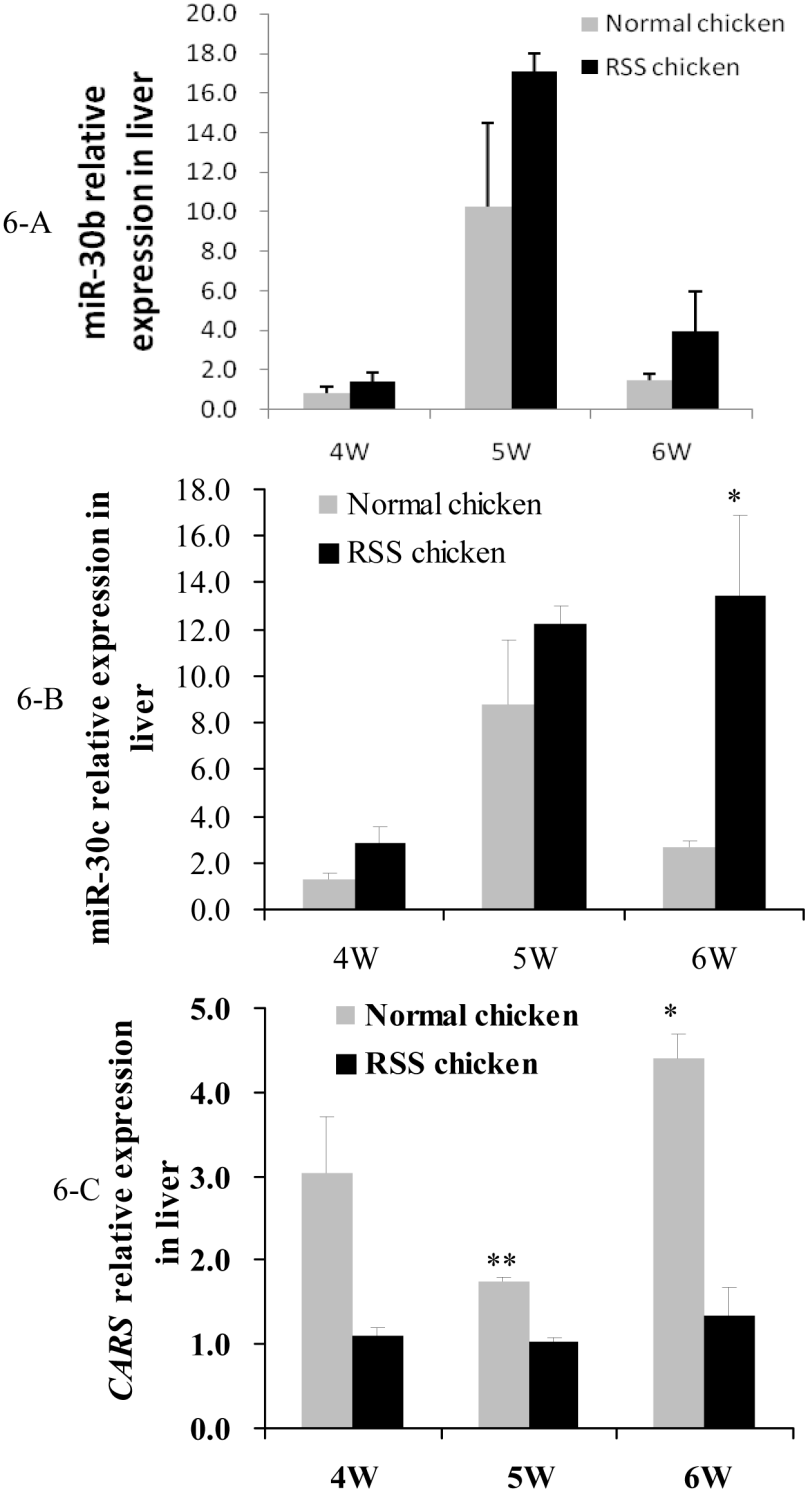
Comparison of the miR-30b/c and *CARS* expression in livers of RSS and normal chickens at 4, 5 and 6 w of age. 6-A, comparison of miR-30b expression in livers. 6-B, comparison of miR-30c expression in livers. 6-C, comparison of *CARS* mRNA expression in livers.

We firstly compared the miR-30b/c and *CARS* expression between normal and RSS chicken livers at three time points (Fig 6). qPCR results indicated that miR-30b/c were expressed in a higher level in RSS chicken than in the normal chickens, while *CARS* showed in an inversed trend. The differences of miR-30c and *CARS* between the two types of chickens reached a significant level at 5 or 6 w of age (Fig 6-B and 6-C) (*P* < 0.05). P value of miR-30b at 6 w of age was 0.08, very close to the statistically significant level (Fig 6-A).

**Fig 7:**
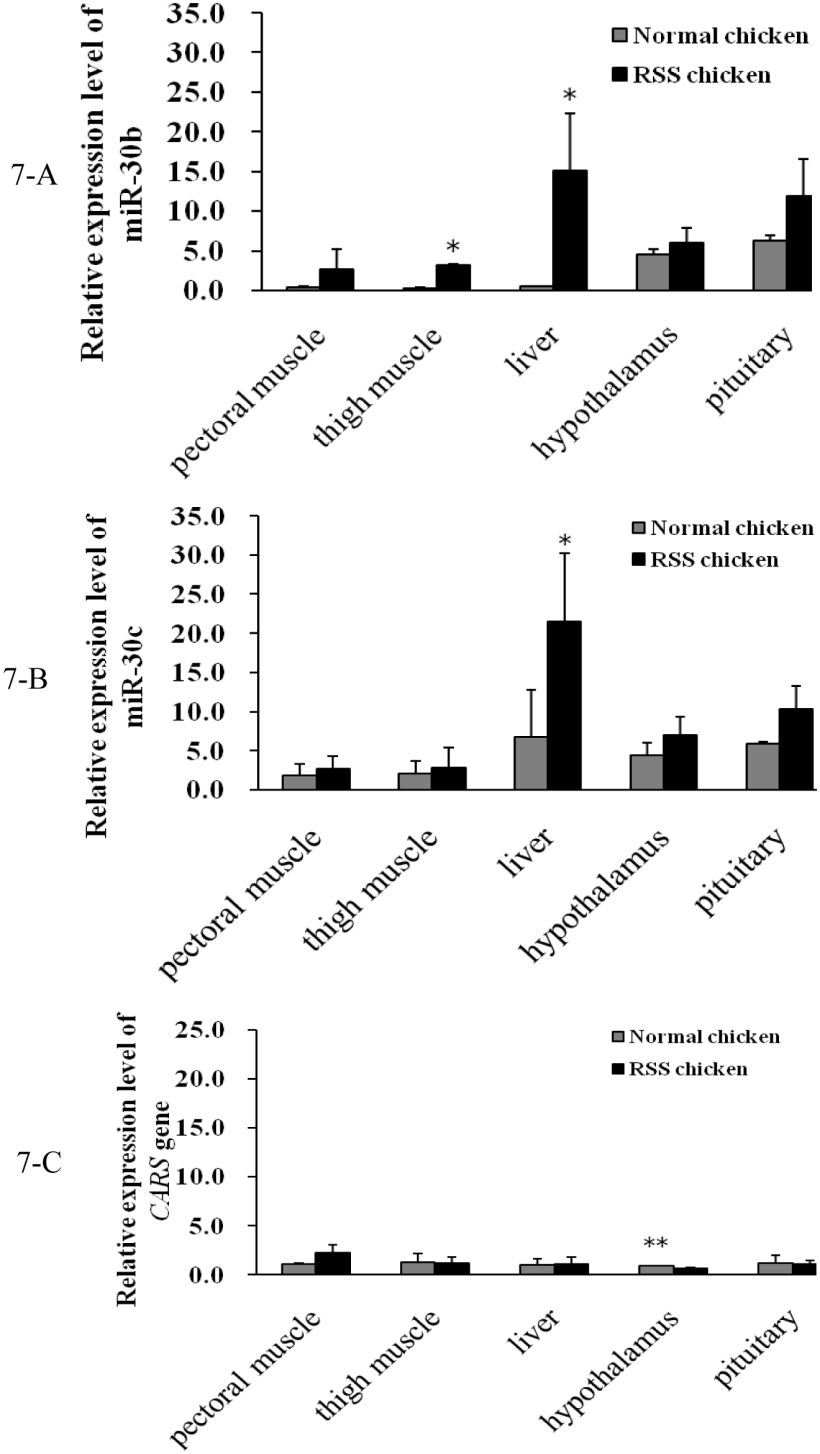
Comparisons of *CARS* mRNA and miR-30b/c in various tissues between RSS and normal chickens at 7 w of age. 7-A, miR-30b expression profile comparison; 7-B, miR-30c expression profile comparison; 7-C, *CARS* mRNA expression profile comparison. “*” represents miR-30b/c or *CARS* was differentially expressed between RSS and normal chickens (*P* < 0.5).

**Fig 8:**
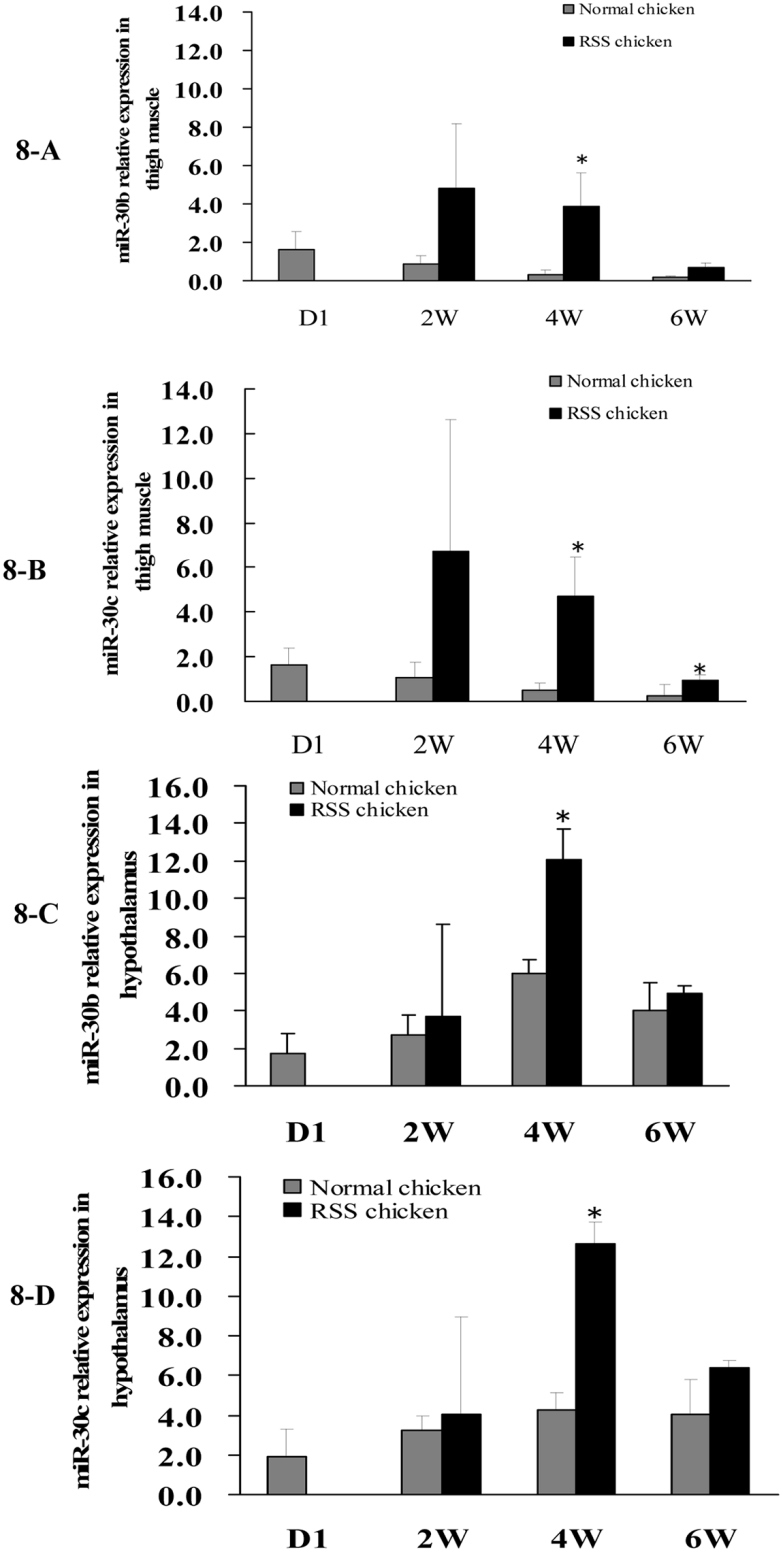
Comparison of miR-30b/30c expression in thigh muscles and hypothalami of RSS and normal chickens at day 1 (D1), 2, 4 and 6 w of age. Quantitative RT-PCR assays of miR-30b and miR-30c expression using total RNA isolated from the chicken thigh muscle and hypothalamus at day1 (D1), 2, 4 and 6 w of age. “*” represents miR-30b or c was differentially expressed between RSS and normal chickens (*P* < 0.5).

**Fig 9:**
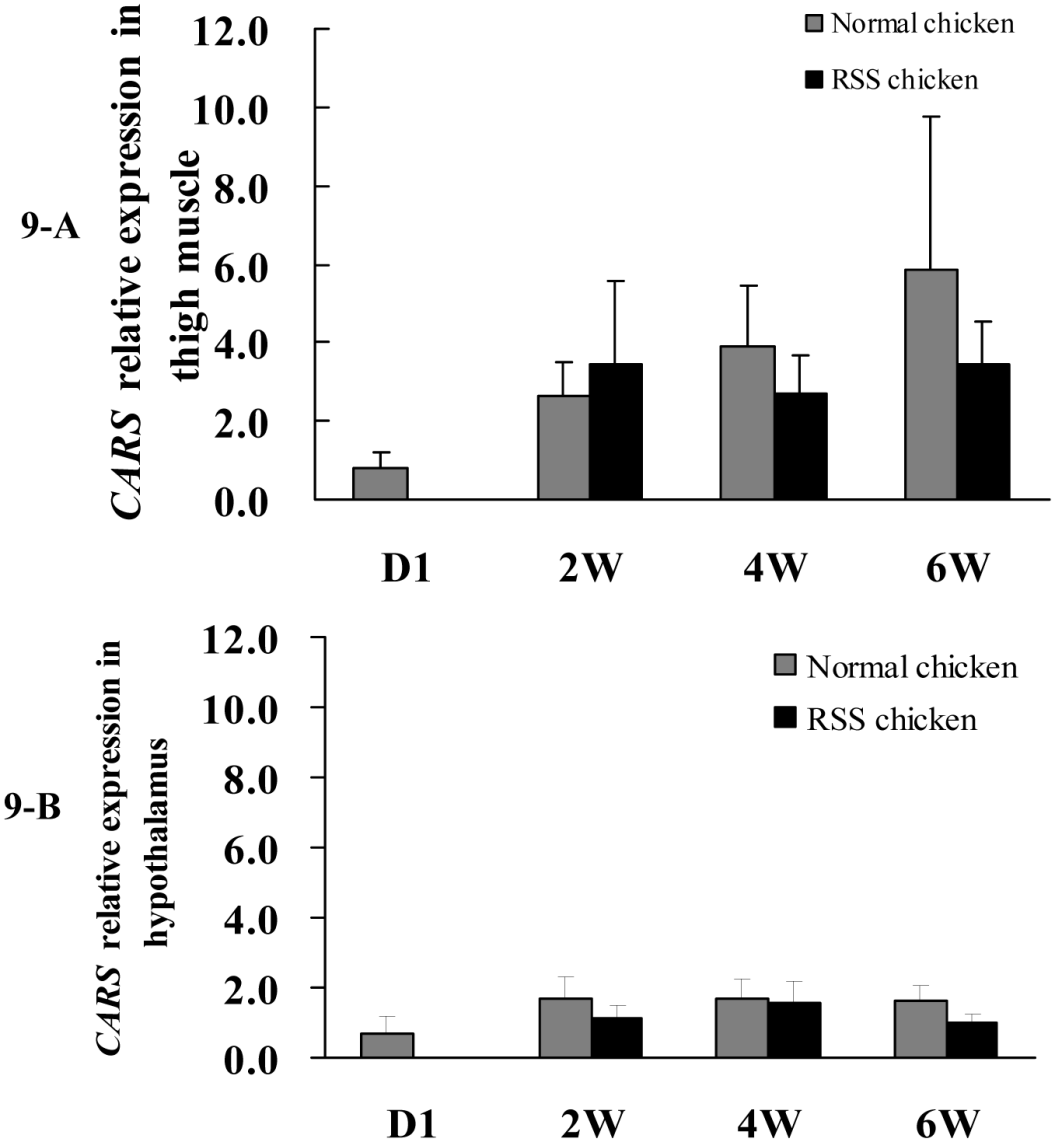
Comparison of *CARS* gene expression in thigh muscles and hypothalami of RSS and normal chickens at day 1 (D1), 2, 4 and 6 w of age. Quantitative RT-PCR assays of *CARS* gene expression using total RNA isolated from the chicken thigh muscle and hypothalamus at day1 (D1), 2, 4 and 6 w of age.

Secondly, we detected the miR-30b/c and *CARS* expression in pectoral muscle, thigh muscle, liver, hypothalamus and pituitary of chicken at 7 w of age and found that miR-30b in thigh muscles and *CARS* in hypothalami were differentially expressed between RSS and normal chickens (Fig 7). Hypothalamic-pituitary system can regulate hormone secretion and further affect animal growth. In addition, thigh muscle weight was an important economic trait in livestock production. Subsequently, qPCR was conducted to compare the expression of miR-30b/c and *CARS* in thigh muscle and hypothalami between RSS and normal chickens at various developmental stages (1 d, 2w, 4wand 6w of age). From 2 to 6 w, miR-30b/c expression was increased in the thigh muscles and hypothalami of RSS chickens compared with normal chickens, and it is notable that significant differences (*P* < 0.05) appeared at 4 w of age (Fig 8). *CARS* expression varied inversely with miR-30b/c expression at 4 w and 6 w of age, although the differences did not attain to statistical significance (*P* > 0.05) (Fig 9).

### 2.8. Variation analysis of 3’UTR of *CARS* gene

We hypothesized that *CARS* gene variation most likely evened the expression differences between RSS and normal chickens. The 3’UTR of the *CARS* gene was amplified to detect variations in RSS or normal chicken DNA samples. However, the nucleotide sequence alignments indicated no nucleotide mutations in the amplified fragment.

### 2.9. Western blot analysis CARS protein in thigh muscle

**Fig 10:**
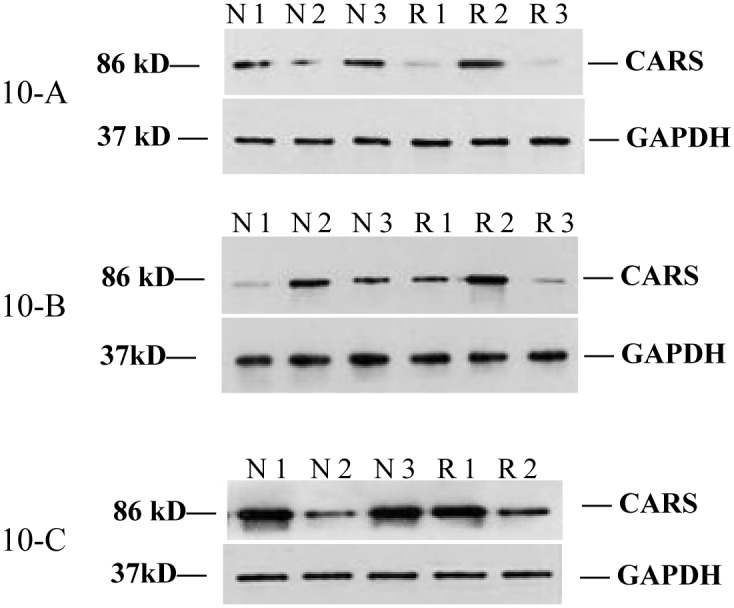
Comparison of CARS protein in thigh muscle between RSS and normal chicken at 2, 4 and week 6. Western blot was conducted to monitor CARS protein differentiation between RSS and normal chickens at 2 (10-A), 4 (10-B) and 6 (10-C) w of age. N1–N3 were normal chickens while R1–R3 were RSS individuals.

Both in normal and in RSS chicken, CARS protein showed an increasing trend along with chicken development. But no obvious differences were observed in thigh muscles as compared normal with RSS chickens at the same stage because of individual differences (Fig 10).

## Discussion

An increasing number of studies indicate that broiler strains differ in susceptibility to infectious diseases, most likely due to their genetic differences [9–11]. In this study, we found 6% RSS chickens exhibiting no pathological changes except for lower body weight. And further we observed significant differences in mRNA and miRNA profiles between RSS and normal chickens. qPCR results of the randomly selected miRNAs and mRNAs displayed good consistency with deep sequencing. In total, 1,159 genes were differentially expressed according to the DGE sequence. Of particular note, six genes (*LOC415662*, *C1orf78*, *ZZEF1*, *CEBPA*, *LOC418836* and *FOXP2*) were specifically expressed in RSS chickens, whereas 5 genes (*CINP*, *MYH3*, *EEF1A2*, *MAP3K13* and *IMMP1L)* were exclusively detected in the livers of normal chickens.

*MAP3K7IP2*, also known as TAB2, is a member of the IL-1 signal transduction pathway, which is a central regulator of immune and inflammatory responses. Moreover, TAB2 is also involved in NF-kappaB activation in the TCR signaling pathway, which plays a key role in the adaptive immune response [23, 24]. Previous cDNA microarray results indicated that immune-related genes were differentially expressed in the intestines of malabsorption syndrome broilers compared with control [10]. Malabsorption syndrome broilers exhibited growth retardation and weight gain depression, and these symptoms are similar to those observed in RSS chickens. Additionally, intestinal cell-mediated immunity also plays a crucial role in broiler development [25]. In short, immunity plays important roles in chicken growth and could be involved in RSS in chickens.

Pathway analysis revealed that the aforementioned genes were involved in 7 pathways at a significant level. It was notable that the enriched pathways were mainly related to energy metabolism, including oxidative phosphorylation, pyruvate metabolism, fatty acid metabolism and primary bile acid biosynthesis pathways. Previous comparative analyses indicated that meat-type chickens, which display a very rapid growth rate, have a higher oxidative phosphorylation efficiency compared with laying-type chickens [26]. In the current study, most genes in the oxidative phosphorylation pathway, such as NADH dehydrogenase (ubiquinone) 1 alpha subcomplex family genes, are expressed at lower levels in RSS chickens compared with normal chickens. Moreover, chickens displaying pale bird syndrome symptoms exhibit a substantial reduction in utilized dietary energy and absorbed lipids compared with normal birds from the commercial farm [27], thereby suggesting that energy metabolism is important in chicken growth.

Recently, antisense transcripts, especially long noncoding RNAs, have attracted increasing attention. Natural antisense transcripts have been identified in diverse organisms, such as human, mouse, chicken andzebrafish [28]. In the current study, the antisense transcripts were widely expressed in chicken liver. The previous known antisense transcripts of bFGF, IGF-II and programmed cell death 2 (*Pdcd2*) genes were also detected in our analysis, indicating that our sequencing results were reliable. Four predicted pathways of the genes with antisense transcripts were differentially compared in RSS and normal chickens. The amino acyl-tRNA biosynthesis pathway, which is specifically enriched in RSS chickens, regulates gene expression and amino acid biosynthesis [29]. The other three pathways (DNA replication, RNA degradation and ubiquitin-mediated proteolysis) specifically observed in normal chickens participate in numerous functions. The four pathways are important in animal development, suggesting that antisense transcripts could function in RSS chicken development. Notably, antisense tags of suppressor of cytokine signaling 3 (SOCS3); myosin, heavy chain 9, non-muscle (MYH9); and inhibitor of growth family member 1 (ING1) genes were also detected with lower abundance. These genes play key roles in the animal growth axis [13]. The role of these antisense tags in regulating chicken growth should be further experimentally verified and studied. Identification of the complicated regulation mechanisms among natural antisense transcripts, miRNAs and mRNAs in animal growth and human health is a great challenge. The latest results on antisense transcription provide an important clue; through an RNA-RNA pairing interaction, the PTENpg antisense transcript affects PTEN protein output by altering PTENpg stability and miRNA sponge activity [30].

Twenty-two differentially expressed miRNAs from liver were identified from Solexa sequencing results. The integration of global profiling of miRNAs and mRNA expression potentially provides a unique opportunity in understanding animal growth [31, 32]. Gga-miR-30b and gga-miR-30c share the same seed regions and belong to the gga-miR-30 family. Members of the miR-30 family function in osteoblast differentiation, adipogenic differentiation, angiogenesis, myocardial matrix remodeling and cell apoptosis by targeting various pathways [18–22, 33]. In this study, gga-miR-30b/c expression was increased in liver in RSS chickens compared with normal chickens. The *CARS* gene was proven as the target of miR-30b/c. gga-miR-30b/c and *CARS* in liver showed inversed expression trends, especially at age of 6w (*P* < 0.05). Also, gga-miR-30b/c expression was increased in the thigh muscle and hypothalamus in RSS chickens compared with normal chickens at the indicated time points. The differences achieved statistical significance at 4 w of age when the maximum gap of body weight was observed between RSS and control chickens [10]. But in the two tissues, the *CARS* mRNA and protein expression did not demonstrate marked differences. In short, abnormal expression of gga-miR-30b/c and *CARS* might lead to RSS chickens by affecting liver metabolism and function.

In addition, variation analysis indicates that no mutations were present in the 3’UTR of this gene. Recent studies showed that the post-transcription regulation mechanism was far more complicated than we expected, and increased dark matters, such as long noncoding RNA, have been confirmed to function in growth and health [34]. Hence, further studies are needed to clarify the role of miR-30b/c in regulating muscle development.

## Materials and Methods

### 4.1. Ethics statement

The Animal Care Committee of South China Agricultural University (Guangzhou, People's Republic of China) approved this study (approval number SCAU#0017). Animals involved in this study were humanely sacrificed as necessary to ameliorate suffering.

### 4.2. Sample collection

Four hundred female RSS yellow-feathered broiler chickens were dissected at 52 d of age. The ones, exhibiting no obvious pathological changes except for lower body weight, were selected for ALV and REV viruses detection. Five negative RSS chickens and 5 normal healthy individuals were used for Solexa and DGE sequencing.

### 4.3. Liver DNA and RNA extraction

Genomic DNA samples were used for ALV and REV virus detection and isolated from livers by a phenolic extraction protocol. Total RNA was extracted from 5 RSS and 5 normal chicken livers using Trizol (Invitrogen, Carlsbad, CA, USA) following the manufacturer’s protocol. Total RNA was tested via agarose electrophoresis, Qubit-fluorometer and Agilent 2100. The five RNA samples isolated from each type of chicken were pooled with equal amounts from individual chickens.

### 4.4. Solexa sequencing of miRNA and Q-RT-PCR validation

The small RNAs (sRNA) were enriched from chicken livers to construct libraries for Solexa sequencing according to previous report [35]. Briefly, 18–40 base pair long RNA fragments were isolated from total RNA using a Novex 15% TBE-Urea gel (Invitrogen). Then, a 5' and 3' adaptor (Illumina, San Diego, CA, USA) were ligated to purified small RNAs followed by purification of ligation products on a Novex 15% TBE-Urea gel. These ligation products were subsequently reverse transcribed and PCR amplified. The purified DNA fragments were used for sequencing using an Illumina Genome Analyzer at the Beijing Genomics Institute, Shenzhen, China. After removing the adaptor/acceptor sequences, filtering the low quality tags and cleaning up the contamination formed by the adaptor-adaptor ligation, the occurrence of each unique sequence reads was counted as sequence tags. We compared the miRNA expression between two kinds of sample and found out the differentially expressed miRNAs according to previous report [36, 37]. Briefly, we normalized the expression of miRNA in two samples (RSS and normal chicken) to get the expression of transcript per million (TPM-Normal and TPM-RSS) at first. Secondly, we calculated fold-change and *P*-value from the normalized expression. According to previous research, denote the number of unambiguous clean tag from gene A as *x*, as every gene's expression occupies only a small part of the library, the *p*(*x*) is in the Poisson distribution [36, 37].

$$p(X=x)=\frac{e^{-\lambda }\lambda^{x}}{x!}$$

(λ represents the population mean tag number of *x*; The capital X represents random variables for the read counts.)

The probability of gene A expressed equally between two samples can be calculated with:

$$p(Y=y|x)={\left(\frac{N_{2}}{N_{1}}\right)}^{y}\frac{(x+y)!}{x!y!{\left(1+\frac{N_{2}}{N_{1}}\right)}^{\left(x+y+1\right)}}$$

(The total clean tag number of the sample 1 is *N1*, and total clean tag number of sample 2 is *N2*; gene A holds *x* tags in sample1 and *y* tags in sample2; The *x* and *y* are not normalized. The capital Y represents random variables for the read counts)

To compute the confidence intervals, we made use of the cumulative distributions:

$$C(y\leq y_{\text{min}}\left|x)=\sum_{y=0}^{y\leq y_{\text{min}}}p(y|x)\;\text{and}\;D(y\geq y_{\text{max}}\right|x)=\sum_{y\geq y_{\max}}^{\infty }p(y|x)$$

which allow the computation of an interval [*y*_*min*_, *y*_*max*_]_ε_ and serve as a significance test when comparing [36, 37]. The intervals are given for the 95% (2ε = 0.05) and 99% (2ε = 0.01) confidence levels and the *P*-value can be calculated as follows when the transcript count *y* and *x* of one gene between the two samples were compared: Without loss of geneality, assume that *y* > *x*

p−value=2P(Y≥y|x)=∑P(Y=yy∗≥y∗|x)

FDR (False Discovery Rate) is the method to determine the threshold of *P* value in multiple test and analysis through manipulating the FDR value [38]. Assume that we have picked out R differentially expressed genes in which S genes are really show differential expression and the other V genes are false positive. We preseted the FDR to a number no larger than 0.001 according to previous report [38] because we decide that the error ratio "Q = V/R" must stay below a cutoff (e.g. 0.1%).

For sequencing data, fold change was calculated according to the following formula,

Fold change =|log2Ratio| = |log2(TPM-Normal/ TPM-RSS)|

One miRNA will be considered as differentially expressed between two types of samples when *P* < 0.05 and fold change ≥1. The differentially expressed miRNAs were selected to verify the results of Solexa sequencing using a miScript Reverse Transcription Kit and miScript SYBR GreenPCR Kit (Qiagen).

### 4.5. Digital gene expression sequencing of mRNA and Q-RT-PCR validation

DGE sequencing of mRNA was documented previously [28]. Briefly, cDNA was digested with NIaIII to obtain a cDNA fragment from the most 3' CATG to the poly(A)-tail. Subsequently, GEX adapter 1 was ligated to the free 5' end of the RNA, and the construct was digested with MmeI, which cuts 17 bp downstream of the CATG site. PCR was performed with primers to enrich the desired fragments. A preprocessed database of all possible CATG+17nt tag sequences was formed using the chicken genome and transcriptome.

One gene will be considered as differentially expressed between two types of samples when *P* < 0.05 and fold change ≥1. The false discovery rate (FDR) was analyzed according to the method reported by Benjamini Yekutieli [38]. P value and fold change were calculated with formulas listed above. Eight differentially expressed genes were selected to confirm the results of DGE sequencing by Q-RT-PCR (ReverTraAce qPCR RT Kit).

### 4.6. Pathway analysis

Three pathway annotation tools, including DAVID v6.7 (http://david.abcc.ncifcrf.gov/), MAS3.0 (http://bioinfo.capitalbio.com/mas3/) and BGI-Cloud (http://bgiamericas.com/data-analysis/bioinformatics-software/), were used to analyze the differentially expressed genes. Pathways with *P* < 0.05 and FDR ≤ 0.05 were considered as significantly enriched in these genes.

### 4.7. Dual-luciferase activity assay

The wild-type and mutated target sequences of *CARS* 3′UTR predicted as target of gga-miR-30b/c was amplified by PCR from chicken cDNA and inserted into the downstream of luciferase reporter gene in the pmirGLO vector (Promega Biotech Co., Ltd., China) (pmirGLO-CARS). The pri-miR-30b/c and the flanking sequences, obtained by PCR from chicken DNA, were inserted into pCDNA3.1+ expression vector (Invitrogen) and subsequently were described as pCDNA3.1-miR-30b/c. pmirGLO-CARS and pCDNA3.1-miR-30b/c plasmids were cotransfected into chick DF-1 cell lines with lipofectamine 3000 reagent (Invitrogen). The relative luciferase activity was detected 36 hours later according to the manufacturing protocol of dual luciferase reporter gene assay kit (Promega Biotech Co., Ltd., China).

### 4.8. Western blot

Leg muscle tissues were collected in lysis buffer (50 mM Tris-HCl, pH 7.5, 150 mMNaCl, 1% Triton X-100, and 0.25% sodium deoxycholate) and were sonicated to shear DNA and reduce sample viscosity. Protein concentration was measured by BCA Protein Assay Kit (Thermo Scientific Pierce, USA). Samples were run on a 10% PAGE gel and transferred onto polyvinylidene fluoride membranes. After blocked in 5% nonfat dry milk in TPBS (0.1% Tween 20 in PBS) for 1 h, membranes were incubated with monoclonal anti-CARS antibody (ab83256, Abcam, SA, USA) overnight at 4°C. After five washes in 5% milk/TPBS 5 min each, membranes were incubated in goat anti-rabbit IgG conjugated with horseradish perioxidase for 1 h followed by two washes in 5% nonfat milk in TPBS, TPBS and PBS 5 min each, respectively. The signals were developed in BeyoECL Plus (Beyotime Institude of Biotech, China). The protein levels were normalized to GAPDH.

### 4.9. Variation analysis of *CARS* gene

PCR and sequencing methods were conducted to analyze variations in the 3’UTR of *CARS* (GenBank accession No. NC_006092) gene.

### 4.10. Statistical analysis

T-test was used to make miR-30b/c and *CARS* temporal and spatial expression comparisons between RSS and normal chickens.

## Supporting Information

S1 Table Differentially expressed genes between RSS and normal chickens. (XLSX)
(XLSX)

S2 Table The differentially expressed genes in the enriched pathways. (DOCX)
(DOCX)

S3 Table Primers for REV and ALV viruses detection. (DOCX)
(DOCX)

S4 Table Primers for miRNA Real-time PCR. (DOCX)
(DOCX)

S5 Table Primers for mRNA Real-time PCR. (DOCX)
(DOCX)

S6 Table Primers used for amplifying 3’-UTR of *CARS* and pri-miR-30b/c. (DOCX)
(DOCX)

S7 Table Primers used for variation analysis of *CARS* gene. (DOCX)
(DOCX)
