# Supplementary material for: A Systematic Analysis on mRNA and MicroRNA Expression in Runting and Stunting Chickens
Source: PLoS One. 2015 May 26;10(5):e0127342. doi: 10.1371/journal.pone.0127342 (PMC4444097; doi:10.1371/journal.pone.0127342)
Supplement: S2 Table — (DOCX) (DOCX) [file pone.0127342.s002.docx]

Table S2 The differentially expressed genes in the enriched pathways

| Pathway | Gene |
| --- | --- |
| Oxidative phosphorylation | *NDUFB5, NDUFB6, NDUFB8, NDUFB9, ATP6V1G1, NDUFB1, NDUFB2, ATP6V0C, NDUFS4, ATP5I, TCIRG1, NDUFB10, NDUFA8, NDUFA9, NDUFA7, ATP5F1, ATP6V1D, LOC770190, PPA1, COX6C, SDHA, ATP6V1E1, COX6A1, ATP6V0A2, LOC770879* |
| Pyruvate metabolism | *LOC423347, LDHB, ALDH7A1, AKR1B1, GLO1, HAGHL, LOC418170, ACAT2, ALDH3A2, MDH2, MDH1, PCK1* |
| Primary bile acid biosynthesis | *ACOX2, CYP39A1, CYP7A1, CYP8B, HSD17B4* |
| Spliceosome | *LSM8, SNRPD3, TRA2B, SF3B5, LOC426296, SF3B4, SMNDC1, CTNNBL1, SFRS5, PRPF8, U2AF1, DHX15, RBM25, HSPA8, BCAS2, PRPF3, CDC5L, SFRS1, DDX5, SF3A1, HNRNPU, EIF4A3, SNRPB, SNRPE, PRPF38B* |
| Proteasome | *PSMA2, PSMA1, PSMB1, PSMA6, PSMD12, PSMC3, PSMA4, SHFM1, PSMA3, PSMC1, PSMD3* |
| Fatty acid metabolism | *ALDH7A1, CPT2, ADH5, ACADL, ACAT2, ALDH3A2, ACSL5* |
| Metabolism of xenobiotics by cytochrome P450 | *CYP3A37, ADH5, EPHX1, LOC421447, LOC396380, UGT1A1, CYP3A80* |
