# Supplementary material for: A Systematic Analysis on mRNA and MicroRNA Expression in Runting and Stunting Chickens
Source: PLoS One. 2015 May 26;10(5):e0127342. doi: 10.1371/journal.pone.0127342 (PMC4444097; doi:10.1371/journal.pone.0127342)
Supplement: S3 Table — (DOCX) (DOCX) [file pone.0127342.s003.docx]

Table S3 Primers for REV and ALV viruses detection

| Primer | Sequence | Tm(℃) | Fragment size (bp) |
| --- | --- | --- | --- |
| REV-F | 5’CATACTGAGCCAATGGTT 3’ | 56 | 300 |
| REV-R | 5’AATGTTGTACMGAARTWCT 3’ |  |  |
| ALV-F | 5’GGATGAGGTGACTAAGAAAG 3’ | 52 | 545 |
| ALV-R | 5’CGAACCAAAGGTAACACACG 3’ |  |  |
