# Supplementary material for: A Systematic Analysis on mRNA and MicroRNA Expression in Runting and Stunting Chickens
Source: PLoS One. 2015 May 26;10(5):e0127342. doi: 10.1371/journal.pone.0127342 (PMC4444097; doi:10.1371/journal.pone.0127342)
Supplement: S4 Table — (DOCX) (DOCX) [file pone.0127342.s004.docx]

Table S4 Primers for miRNA Real-time PCR

| Primer | Sequence | Tm(℃) | Fragment size (bp) |
| --- | --- | --- | --- |
| gga-miR-215F | 5' ATGACCTATGAATTGACAGACAAA 3' | 51 | 79 |
| gga-miR-221F | 5’ TACATTGTCTGCTGGGTTTCAA 3’ | 55 | 78 |
| gga-miR-30bF | 5' GTAAACATCCTACACTCAGCTAAA 3' | 55 | 78 |
| gga-miR-30cF | 5’ AACATCCTACACTCTCAGCTAA 3’ | 55 | 76 |
| gga-miR-375F | 5' GTTCGTTCGGCTCGCGTTAAAA 3' | 51 | 75 |
| gga-5S rRNA-F | 5’ ACCGGGTGCTGTAGGCTTAA 3’ | 55 | 73 |
| Universal primer | Provided by miScript SYBR® GreenPCR Kit (Qiagen) | | |
