# Supplementary material for: A Systematic Analysis on mRNA and MicroRNA Expression in Runting and Stunting Chickens
Source: PLoS One. 2015 May 26;10(5):e0127342. doi: 10.1371/journal.pone.0127342 (PMC4444097; doi:10.1371/journal.pone.0127342)
Supplement: S5 Table — (DOCX) (DOCX) [file pone.0127342.s005.docx]

Table S5 Primers for mRNA Real-time PCR

| Primer | Sequence | Gene | Tm(℃) | Fragment size (bp) | Genbank Accession No. |
| --- | --- | --- | --- | --- | --- |
| P1-F  P1-R | 5'TGCCCTGCTGAGCGAGTG 3'  5'CTCCCCTTCTGCTGGCTGTT 3' | *CKAP2L* | 62 | 260 | XM_424317.2 |
| P2-F  P2-R | 5'GCATTAGAGGGAGCAAACGTGAT 3'  5'CAATCTGATCTCAAAGGGCATGTT 3' | *TBPL1* | 59 | 257 | NM_204755.1 |
| P3-F  P3-R | 5'GCGGGGTGGTGTTCAAA 3'  5'TCCAGCCTTTTTCAGCACTT 3' | *MAP2K1* | 58 | 234 | NM_001005830.1 |
| P4-F  P4-R | 5'GCGGGTGCTGACGGTGCTG 3'  5'CCCCGGTGTCGTTGCCTATGA 3' | *KDR* | 62 | 270 | NM_001004368.1 |
| P5-F  P5-R | 5'TTCTTCGAGCTCCCACTGATGT 3'  5'AGCAGTCTGTTTGGCATTTGTCT 3' | *CARS* | 61 | 228 | NM_001012583.1 |
| P6-F  P6-R | 5'AACAGCAGCAGCATCATACAG 3'  5'ATTCCATTTTCTCCATCTGTTAC 3' | *MAP3K7IP2* | 61 | 245 | XM_419660.2 |
| P7-F  P7-R | 5'TGCAACATTCTTTTACTTCAACCA 3'  5'GAAGGTGTCACATTTTGGTTTTG 3' | *MAP3K5* | 61 | 213 | XM_419725.2 |
| P8-F  P8-R | 5'GGCCTCCTTAAAGAAAACCATGTC 3'  5'AGGGGCTCCGTGCTAATGATC 3' | *LARP7* | 61 | 247 | XM_420643.2 |
| P9-F  P9-R | 5'CCCCATGCCATCCTCCGTCTG 3'  5'CCTCGGGGCACCTGAACCTCTC 3' | *beta-actin* | 61 | 223 | NM_205518 |
