# Supplementary material for: A Systematic Analysis on mRNA and MicroRNA Expression in Runting and Stunting Chickens
Source: PLoS One. 2015 May 26;10(5):e0127342. doi: 10.1371/journal.pone.0127342 (PMC4444097; doi:10.1371/journal.pone.0127342)
Supplement: S6 Table — (DOCX) (DOCX) [file pone.0127342.s006.docx]

Table S6 Primers used for amplifying 3’-UTR of *CARS* and pri-miR-30b/c

| Gene | Sequence | Restriction enzyme | Tm (℃) | Fragment size (bp) |
| --- | --- | --- | --- | --- |
| *CARS* | F:5'GCGG*TTTAAA*TTAGAAGGCAGGAGTTGG 3'  R:5’ GATC*TCTAGA*GGATCGGTATGCAGGACA 3’ | Dra I  Xba I | 61 | 312 |
| Pri-miR-30b | F:5'ATT*GCGGCCGC*AGTGCTCCAACTTTAC 3'  R:5'GGG*GGGCCC*AGGACACCAGAATGTAC 3' | Not I  Apa I | 54 | 491 |
| Pri-miR-30c | F:5'TAAT*GCGGCCGC*AGTAAGAGTGGCTGTCCT 3'  R:5'CGAT*GGGCCCC*TGCTACCATTTGCGTTT 3' | Not I  Apa I | 60 | 554 |

Note: The underlined sequences were restriction enzyme cutting sites.
