# Supplementary material for: A Systematic Analysis on mRNA and MicroRNA Expression in Runting and Stunting Chickens
Source: PLoS One. 2015 May 26;10(5):e0127342. doi: 10.1371/journal.pone.0127342 (PMC4444097; doi:10.1371/journal.pone.0127342)
Supplement: S7 Table — (DOCX) (DOCX) [file pone.0127342.s007.docx]

Table S7 Primers used for variation analysis of *CARS* gene

| Sequence | Tm(℃) | Fragment size (bp) |
| --- | --- | --- |
| F:5'AGGCCAAAAAACAAACATTACC3' R:5'CACGTGGATTTGCTGACTGAG 3' | 55 | 624 |
